# A novel de novo *SLC26A3* mutation causing congenital chloride diarrhea in a Japanese neonate

**DOI:** 10.1002/mgg3.1505

**Published:** 2020-09-20

**Authors:** Ken‐ichiro Konishi, Tatsuki Mizuochi, Hitoshi Honma, Yuri Etani, Kazue Morikawa, Kazuko Wada, Ken Yamamoto

**Affiliations:** ^1^ Department of Pediatrics and Child Health Kurume University School of Medicine Kurume Japan; ^2^ Department of Pediatric Surgery Graduate School of Medicine The University of Tokyo Tokyo Japan; ^3^ Department of Gastroenterology and Endocrinology Osaka Women's and Children's Hospital Osaka Japan; ^4^ Department of Neonatal Medicine Osaka Women's and Children's Hospital Osaka Japan; ^5^ Department of Medical Biochemistry Kurume University School of Medicine Kurume Japan

## Abstract

**Background:**

Congenital chloride diarrhea (CCD) is characterized by persistent chloride (Cl)‐rich diarrhea evident from birth. CCD is a rare autosomal recessive disorder caused by defects in the solute carrier family 26 member 3 (*SLC26A3*) gene, which encodes an intestinal Cl^−^/HCO3^−^, Na^+^‐independent exchanger. Various mutations of *SLC26A3* have been described in CCD. However, no de novo mutations have been found to be responsible for CCD. Here we report the first such occurrence.

**Methods:**

Clinical and laboratory findings during the perinatal period were obtained retrospectively from medical records. Mutations involving *SLC26A3* were detected by Sanger sequencing.

**Results:**

The male infant reported here was delivered at 29 weeks of gestation. Just after birth, he had watery diarrhea without meconium passage. High chloride concentrations in the diarrhea led to a diagnosis of CCD. Direct sequencing of all coding exons in *SLC26A3* including exon‐intron boundaries disclosed 2 compound heterozygous mutations: c.382G>A, p.G128S and c.2063‐1g>t. The c. 2063‐1g>t mutation was confirmed in his mother's DNA, but c.382G>A, p.G128S was absent in both mother and father.

**Conclusion:**

We concluded that c.382G>A, p.G128S represented a de novo mutation of *SLC26A3*, a very rare event in autosomal recessive disorders. To our knowledge, this is the first CCD case involving a de novo novel mutation of *SLC26A3*.

## Graphical abstract

The manuscript describes a Japanese newborn who was diagnosed with congenital chloride diarrhea caused by a novel de novo mutation of *SLC26A3*. De novo mutation in an autosomal recessive disorder may be extremely rare. To our knowledge, this is the first reported case of congenital chloride diarrhea with a de novo novel mutation in *SLC26A3*.

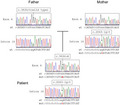

## 1 INTRODUCTION

Congenital chloride (Cl) diarrhea (CCD; MIM# 214700) is characterized by persistent Cl‐rich diarrhea evident from birth. Intrauterine abnormalities such as polyhydramnios, dilated bowel loops, and absence of meconium can simulate intestinal obstruction, resulting in unnecessary surgical exploration. Soon after birth, the profuse diarrhea can be complicated by dehydration, hypochloremia, hypokalemia, metabolic alkalosis, and failure to thrive. Diagnosis of CCD is based upon watery diarrhea that begins soon after birth and contains excessive fecal chloride. Untreated CCD causes early death from impaired renal function and end‐stage renal disease, but early diagnosis and oral salt replacement with sodium Cl (NaCl) and potassium Cl (KCl) allow a favorable long‐term outcome permitting normal growth and development (Konishi et al., 2019; Wedenoja, Hoglund, & Holmberg, 2010).

Congenital chloride diarrhea, a rare autosomal recessive disorder, can result from any of over 100 mutations of the solute carrier family 26 member 3 (*SLC26A3*) gene, which encodes an intestinal Cl^−^/HCO3^−^, Na^+^‐independent exchanger (Hoglund et al., 1998; Konishi et al., 2019; Makela, Kere, Holmberg, & Hoglund, 2002; Wedenoja et al., 2011). Genetic findings in CCD have been reported predominantly from 3 areas with high incidence: Finland, Poland, and Saudi Arabia. In these countries, 3 mutations (c.559G>T, c.951_953del, and c.2024_2026dup) account for 47%–94% of genetically analyzed cases, a distribution believed to reflect founder effects (Hoglund et al., 1998; Makela et al., 2002; Wedenoja et al., 2011). In contrast, great genetic heterogeneity has been reported among approximately 300 CCD patients from other ethnic groups, involving mutations scattered throughout the *SLC26A3* gene (Lechner et al., 2011; Makela et al., 2002). Recently, 13 Japanese CCD patients with *SLC26A3* mutations were identified by a nationwide survey in which we likewise noted diversity among the causative mutations (Konishi et al., 2019). CCD has affected families from Turkey, Japan, and other countries (Doğan et al., 2020; Yoshikawa, Watanabe, Abe, Sato, & Oda, 2000). However, no de novo mutations have been found to be responsible for CCD. Here, we report the first such occurrence.

## 2 MATERIALS AND METHODS

Clinical and laboratory findings during the perinatal period were obtained retrospectively from medical records.

Genomic DNA was isolated from the patient's and parents’ peripheral blood cells for detection of *SLC26A3* mutations. DNA was subjected to PCR using primers described in a previous report (Konishi et al., 2019). These primer sets were designed to amplify 21 exons including the 5′‐untranslated region and the coding regions including exon‐intron boundaries. PCR products were treated with ExoSAP‐IT Express PCR Cleanup Reagents (Thermo Fisher Scientific; Waltham, MA) to inactivate free primers and deoxyribonucleotide triphosphates (dNTPs), and then subjected to sequencing reactions using forward or reverse primers and BigDye Terminator v3.1 (Thermo Fisher Scientific). DNA fragments were purified using Centri‐Sep spin columns (Princeton Separations; Princeton, NJ). Sequencing was carried out with an ABI 3100 Genetic Analyzer (Applied Biosystems; Waltham, MA). Sample sequences were aligned to reference sequences obtained from the University of California, Santa Cruz (UCSC) Genome Bioinformatics website (http://genome.ucsc.edu/index.html) using the ClustalW program (https://www.genome.jp/tools​-bin/clustalw) to identify nucleotide changes. Mutations were numbered according to GenBank Reference Sequence NM_000111. Names of identified variants were assigned following the guidelines of the Human Genome Variation Society (version 2; http://www.hgvs.org/mutno​men/). Chromosomal coordinates were assigned according to the GRCh37/hg19 assembly. The Human Gene Mutation Database (HGMD, January 2015; http://www.hgmd.org/), Sorting Intolerant Form Tolerant (SIFT; http://sift.jcvi.org/), and MutationTaster2 (http://www.mutat​ionta​ster.org/) were used as computational predictive programs. Allele frequency was referred to the UCSC Genome Browser and the Japanese data set of the Human Genetic Variation Database (HGVD; http://www.hgvd.genome.med.kyoto​-u.ac.jp/). American College of Medical Genetics and Genomics (ACMG) interpretation guidelines were followed in assessing pathogenicity of detected variants (Richards et al., 2015). The genetic analysis protocol was approved by the ethics committees at Kurume University. Written informed consent was obtained from the patient's parents.

### 2.1 Case presentation and genetic analysis results

A 25‐year‐old healthy Japanese primigravida was referred by a maternity hospital. The fetus was suspected to have either CCD or congenital intestinal atresia (CIA) based on the fetal ultrasonographic findings of polyhydramnios and dilated bowel loops. After fetal heart monitoring detected frequent severe variable decelerations, a male infant was delivered by cesarean section at 29 weeks of gestation. Birth weight was 1434 g; Apgar scores were 7 and 8 at 1 and 5 minutes, respectively. He was the first child of non‐consanguineous healthy parents. Admission to the neonatal intensive care unit was required by very low birth weight and suspected CCD. The newborn's abdomen was distended, and watery diarrhea lacking meconium passed soon after delivery. The stool had a high concentration of Cl (147 mmol/L; reference value, <90 mmol/L).(Holmberg, Perheentupa, & Launiala, 1975) Serum electrolytes on admission were Na, 139 mmol/L; K, 4.0 mmol/L; and Cl, 101 mmol/L. Results of venous blood gas analysis were pH, 7.363; pCO2, 35.6 mmHg; HCO3^−^, 19.7 mmol/L; and base excess, −4.4 mmol/L. Urinary electrolyte values were Na, 38 mmol/L; K, 4.0 mmol/L; and Cl, 37 mmol/L. Abdominal radiography ruled out intestinal obstruction. On the second day of life, he was diagnosed with CCD based on frequent watery diarrhea beginning soon after birth and high fecal chloride (Konishi et al., 2019; Wedenoja et al., 2010). Intravenous and oral replacement therapy with NaCl and KCl was administered. With treatment, serum electrolytes were maintained largely within the normal range (Table 1), and general condition was good. Ninety days after birth, weighing 3168 g, he was discharged home.

**TABLE 1:** Serum electrolyte concentrations before and after treatment

Days after birth	0	3	7	19	57	85
Serum Na (mmol/L)	139	140	135	138	140	137
Serum K (mmol /L)	4.0	3.6	4.0	3.7	3.6	3.7
Serum Cl (mmol/L)	101	102	102	96	96	93
Treatment	Pretreatment	Infusion of electrolytes including Na, K, and Cl	Infusion of electrolytes including Na, K, and Cl	Oral NaCl and KCl	Oral NaCl and KCl	Oral NaCl and KCl

Note:

Na, sodium; K, potassium; Cl, chloride.

**FIGURE 1:**
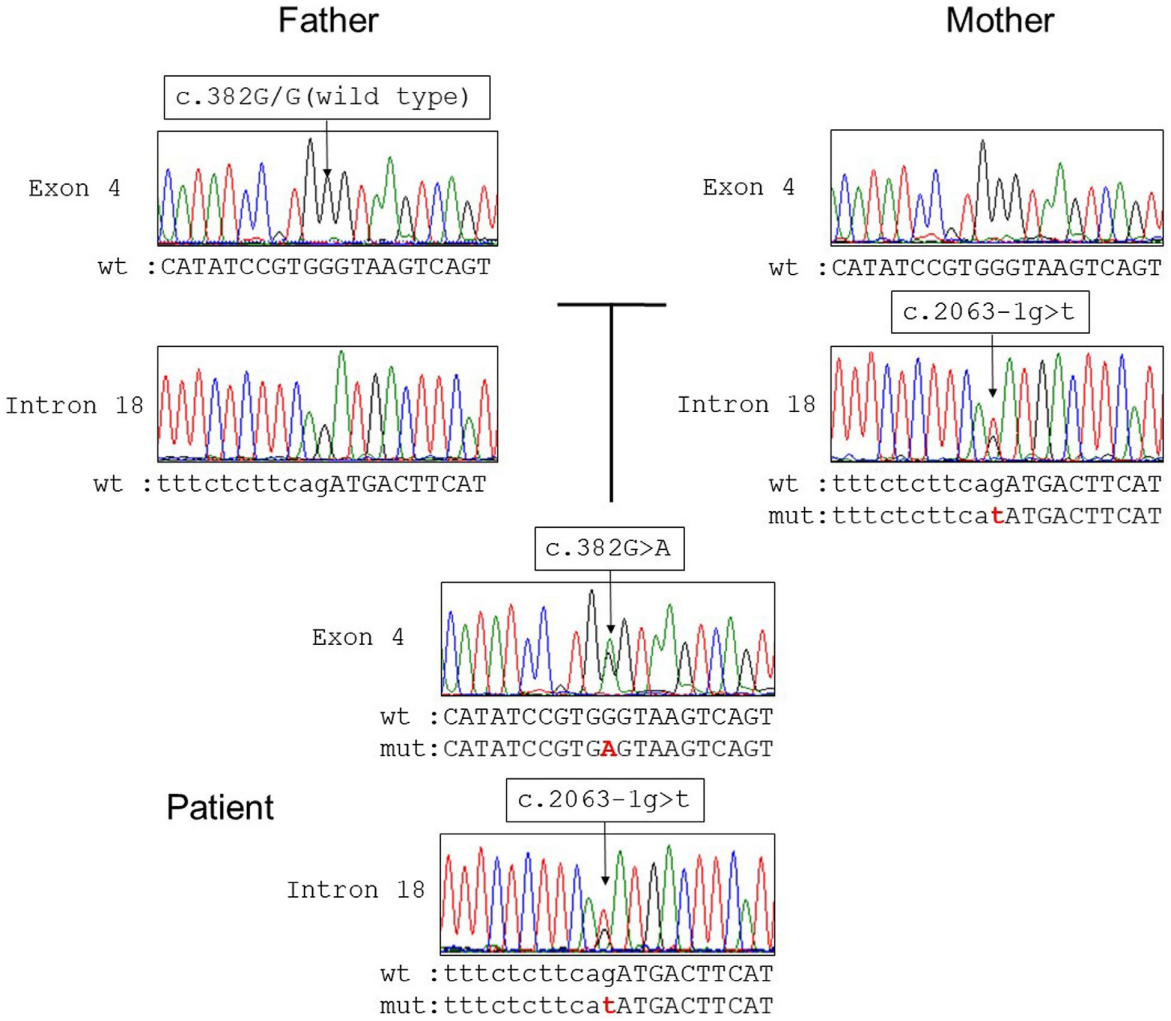
Sanger sequencing showing *SLC26A3* mutations detected in the patient and his mother. The patient was heterozygous for 2 pathogenic mutations: c.382G>A, p.G128S, and c.2063‐1g>t. While c.2063‐1g>t was confirmed in his mother, c.382G>A, p.G128S was not present in either parent

**FIGURE 2:**
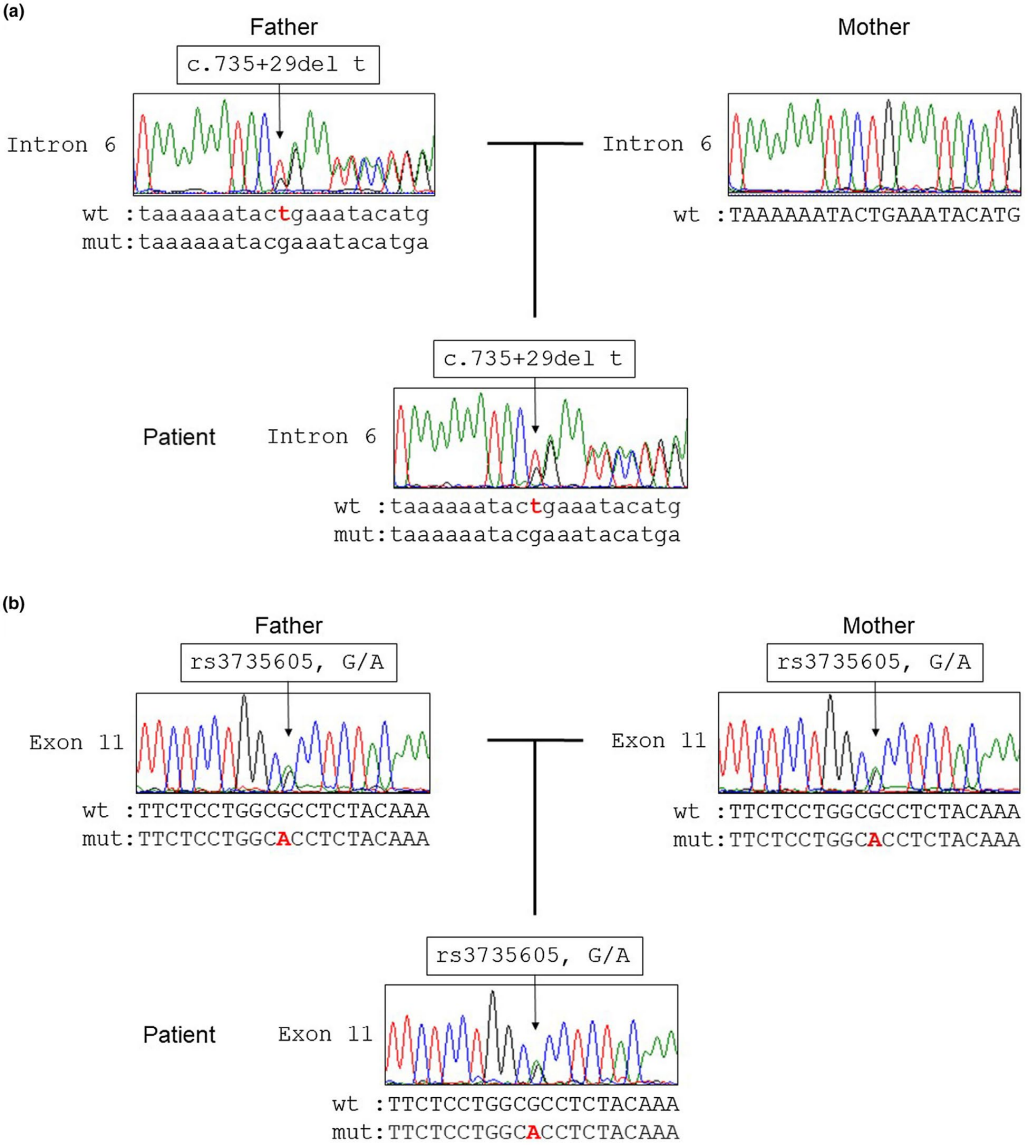
Evidence of paternity for this patient. (a) c.735+29del t was identified in both the patient and his father, though not in his mother. (b) rs3735605 was identified in the patient and both parents

We screened all coding exons of *SLC26A3* (NCBI ref: NM_000111) including exon‐intron boundaries by direct sequencing. Genetic analysis detected compound heterozygosity of *SLC26A3*; the 2 relevant mutations were c.382G>A, p.G128S and c.2063‐1g>t. The c.2063‐1g>t mutation also was present in the mother's DNA, but c.382G>A, p.G128S was absent from both parents’ samples (Figure 1). Confounding paternity issues were ruled out by the presence of the rare nonpathogenic variant c.735+29del t in both the patient and his father (Figure 2A). Further, the common variant c.1299G>A (rs3735605) was identified in both patient and parents (Figure 2B), and the ABO blood groups of the patient, mother, and father respectively were types B, B, and O. Therefore, c.382G>A, p.G128S most likely represents a de novo mutation of *SLC26A3*.

## 3 DISCUSSION

In this study we report a Japanese neonate with CCD caused by *SLC26A3* mutations including a novel de novo missense variant.

Soon after birth, which often is premature, children with CCD exhibit profuse watery diarrhea and often consequent dehydration. Hypochloremia and hypokalemia are characteristic, and metabolic alkalosis is likely in the absence of electrolyte replacement. Failure to thrive can come to dominate the clinical picture. Diagnosis of CCD involves recognition of watery diarrhea soon after birth and detection of excessive fecal chloride. Differential diagnoses for CCD include CIA, cystic fibrosis, and Bartter syndrome. Misdiagnosis of Bartter syndrome becomes likely if watery diarrhea is unnoticed or mistaken for urine. Untreated CCD causes early death as impaired renal function progresses to nephrocalcinosis and end‐stage renal disease, but early initiation of salt replacement with NaCl and KCl can be continued orally to permit normal long‐term growth and development. In our Japanese study, long‐term outcome compared favorably with some previous reports, reflecting early diagnosis and intervention (Hihnala et al., 2006; Konishi et al., 2019). Pediatricians, obstetricians, and pediatric surgeons should be aware of CCD as well as its differential diagnostic alternatives including CIA, cystic fibrosis, and Bartter syndrome (Konishi et al., 2019).

The patient was heterozygous for c.382G>A, p.G128S and c.2063‐1g>t; both were classified as pathogenic according to ACMG standards and guidelines (Richards et al., 2015). *SLC26A3* mutations have been identified in a large majority of known Japanese CCD patients (93%), (Konishi et al., 2019) demonstrating the usefulness of genetic analysis of *SLC26A3* in definitive diagnosis of CCD.

The c.2063‐1g>t mutation was previously reported in Japan, occurring in at least one allele of seven patients in our series of 13 (54%) (Konishi et al., 2019). In addition, the c.2063‐1g>t mutation was identified in all reported Korean patients but in no reported Finnish, Polish, or Arab patients (Hong et al., 2013; Wedenoja et al., 2011). This suggests that c.2063‐1g>t may represent a founder mutation in East Asia (Hong et al., 2013; Konishi et al., 2019).

Because c.382G>A, p.G128S was not previously registered in genomic databases including the UCSC genome browser, Human Genetic Variation Database, and Human Gene Mutation Database, we concluded that it most likely represents a novel missense variant. *SLC26A3*, previously known as downregulated in adenoma (DRA), is located on chromosome 7q31 and consists of 21 exons. Its product is an apical transmembrane protein with 764 amino acids, including 14 probable hydrophobic membrane‐spanning domains and a cytoplasmic COOH‐terminal tail. The tail possesses two protein interaction motifs: a sulfate transporter and anti‐sigma antagonist (STAS) domain including amino acids 525 to 720, and a post‐synaptic density 95/discs large/zona occlusion (PDZ)‐binding domain including amino acids 762–764 (Holmberg, Perheentupa, Launiala, & Hallman, 1977; Wedenoja et al., 2011). Among 107 previously reported mutations, 72 are known to be located in the transmembrane domain and 31 in the STAS domain (Konishi et al., 2019). The c.382G>A, p.G128S mutation is located in the transmembrane domain, which is essential to the anion‐exchange functions of the SLC26A3 protein. While c.382G>A, p.G128S was detected in neither the patient's mother nor the father, paternity issues were essentially ruled out by the presence of a very rare nonpathogenic variant, c.735+29del t, in both the patient and his father. A shared, more common single nucleotide polymorphism, c.1299G>A (rs3735605), and also blood types, additionally support paternity even in the absence of genetic analyses specifically focused on that question. Accordingly, c.382G>A, p.G128S most likely arose from de novo mutation in *SLC26A3*.

Three recently published trio‐based exome sequencing studies, each including several thousand cases, did not include a single instance of an autosomal recessive disorder where one of the causative alleles was a de novo mutation (Kong et al., 2012; Retterer et al., 2016; Yang et al., 2014). Retterer et al. (2016) reported exome sequencing in more than 3000 cases and Yang et al. (2014) in more than 2000 cases; together these studies included more than 400 autosomal recessive molecular diagnoses without a single case of a de novo mutation contributing to the genetic etiology. Therefore, de novo mutation contributing to an autosomal recessive disorder would be extremely rare. This should reassure the parents of the present CCD patient about having additional children.

In conclusion, to our knowledge, the present CCD case is likely to involve a novel de novo mutation in *SLC26A3*, and illustrates the value of genetic analysis in definitive diagnosis of CCD.

## CONFLICT OF INTEREST

We have no conflict of interest.

## AUTHOR CONTRIBUTIONS

KK, TM, and KY conceptualized and designed the study, collected data, carried out the initial analyses, participated in genetic analysis, drafted the initial manuscript, and reviewed and revised the manuscript. HH, YE, KM, and KW collected data and reviewed and revised the manuscript. All authors approved the final manuscript as submitted and agree to be accountable for all aspects of the work.
